# Digital Twin in Managing Hypertension Among People With Type 2 Diabetes: 1-Year Randomized Controlled Trial

**DOI:** 10.1016/j.jacadv.2024.101172

**Published:** 2024-08-14

**Authors:** Paramesh Shamanna, Shashank Joshi, Mala Dharmalingam, Arun Vadavi, Ashok Keshavamurthy, Lisa Shah, Shambo Samrat Samajdar, Jeffrey I. Mechanick

**Affiliations:** a Department of Diabetes, Bangalore Diabetes Centre, Bangalore, Karnataka, India; b Department of Diabetology and Endocrinology, Lilavati Hospital and Research Center, Mumbai, India; c MS Ramaiah Medical College, Bangalore Endocrinology & Diabetes Research Centre, Bangalore, Karnataka, India; d Department of Diabetes, Sudha Prevention Centre, Bangalore, Karnataka, India; e Department of Diabetes, Chandana Clinic, Bangalore, Karnataka, India; f Twin Health, Mountain View, California; g Department of Clinical and Experimental Pharmacology, Calcutta School of Tropical Medicine, Kolkata, India; h The Marie-Josee and Henry R. Kravis Center for Cardiovascular Health at Mount Sinai Fuster Heart Hospital, New York City, New York, USA

**Keywords:** artificial intelligence, digital twin, hypertension remission, internet of things, normoalbuminuria, normotension

## Abstract

**Background:**

Digital twin (DT)-guided lifestyle changes induce type 2 diabetes (T2D) remission but effects on hypertension (HTN) in this population are unknown.

**Objectives:**

The purpose of this study was to assess effects of DT vs standard of care (SC) on blood pressure (BP), anti-HTN medication, HTN remission, and microalbuminuria in participants with T2D.

**Methods:**

This is a secondary analysis of a randomized controlled trial in India of 319 participants with T2D. Participants were randomized to DT group (N = 233), which used artificial intelligence-enabled DT technology, or SC group (N = 86). A Home Blood Pressure Monitoring system guided anti-HTN medication adjustments. BP, anti-HTN medications, HTN remission rates, and microalbuminuria were compared between groups.

**Results:**

Among the 319 participants, 44 in DT and 15 in SC group were on anti-HTN medications, totaling 59 (18.4%) participants. DT group achieved significant reductions in systolic blood pressure (−7.6 vs −3.2 mm Hg; *P* < 0.007) and diastolic blood pressure (−4.3 vs −2.2 mm Hg; *P* = 0.046) after 1 year compared with SC group. 68.2% of DT group remained off anti-HTN medications compared to none in SC group. Among participants with HTN, DT subgroup achieved higher rates of normotension (40.9% vs 6.7%; *P* = 0.0009) and HTN remission (50% vs 0%; *P* < 0.0001) than SC subgroup. DT group had a higher rate of achieving normoalbuminuria (92.4% vs 83.1%; *P* = 0.018) at 1 year compared with SC group.

**Conclusions:**

Artificial intelligence -enabled DT technology is more effective than SC in reducing BP and anti-HTN medications and inducing HTN remission and normoalbuminuria in participants with HTN and T2D. (A Novel WholeBody Digital Twin Enabled Precision Treatment for Reversing Diabetes; CTRI/2020/08/027072)

Hypertension (HTN) is commonly found in individuals with type 2 diabetes (T2D),^1^ with about 74% of patients with T2D having high blood pressure (BP) or taking HTN medication.^2^ The co-occurrence of HTN and T2D raises the risk of cardiovascular diseases, diabetic nephropathy, and retinopathy, making management challenging.^3^^,^^4^ Effective BP control is essential for reducing these complications.^5^ Studies like the Hypertension Optimal Treatment Study and UK Prospective Diabetes Study emphasize the importance of strict BP control to prevent complications.^5^, ^6^, ^7^ However, many patients do not reach BP targets with 1 medication alone,^8^ often requiring multiple drugs.^9^, ^10^, ^11^, ^12^ Despite greater treatment rates, HTN remains widespread, underlining the need for more efficient treatment approaches.^13^

The interactions among adiposity, dysglycemia, HTN, and cardiovascular disease harbor insulin resistance as a common denominator with networking effects well established with epidemiological and mechanistic study.^14^^,^^15^ Weight loss independently reduces BP and improves glucose and lipid levels. A 1-kg weight loss can decrease mean arterial BP by 1 mm Hg.^16^ Dietary sodium restriction also effectively lowers BP in essential HTN.^17^ Moderate-intensity physical activity such as brisk walking can lower BP.^18^ Hyperglycemia and HTN lead to endothelial dysfunction and cardiovascular disease.

The Digital Twin (DT) intervention, leveraging artificial intelligence (AI), wearable sensors, and health coaching, not only fosters T2D remission through tailored lifestyle adjustments but also effectively reduces body weight and insulin resistance.^19^ This, in turn, contributes to improved HTN management, with digital nudges playing a pivotal role in encouraging personalized dietary modifications and physical activity changes for better health outcomes. Managing T2D through diet and exercise improves glycemic parameters and cardiovascular risk factors like HTN and hypercholesterolemia. Controlling weight with dietary restrictions is crucial to reduce diabetes-related risks.^20^^,^^21^ Ectopic fat, excess adipose tissue in nonstorage locations, contributes to obesity-related vascular diseases and associated risks.^22^ Fat depots include visceral adipose tissues, intrahepatic and intramuscular fats, pericardial fat, perivascular fat, and renal sinus fat.^22^ These depots increase cardiovascular disease risk and inflammation due to obesity and insulin resistance.^23^ Higher body mass index, body circumferences, and visceral fat increase cardiovascular risk.^24^ Lifestyle programs help manage diabetes and associated risks.

We present a secondary analysis of a dataset previously reported for glycemic and liver parameters. This current analysis focuses on BP control, anti-HTN medication use, HTN remission rates, and microalbuminuria, in the setting of an AI-enabled digital therapeutics intervention.

## Methods

### Study Design

This is a multicenter randomized controlled trial with a novel AI-enabled DT intervention for T2D.

### Participants and Eligibility Criteria

Participants aged 18 to 70 years with T2D ≤8 years, normal hepatic/renal function, and smartphone capability were recruited from 4 large hospitals in India. Exclusion criteria include pregnancy/nursing, diabetes other than T2D, past/planned bariatric procedure, or major psychiatric disorder.

### Randomization and Masking

Sample sizes were determined a priori according to an expected difference in hemoglobin A1c (HbA1c) decrease of ≥1% comparing the 2 groups (DT vs standard of care [SC]). In a prior study, the HbA1c decrease at 90 days showed a SD of 1·83.^25^ An α-level of 0·05 and power of 90% (allowing for an approximate 20% dropout rate) was stipulated to detect a statistically significant difference in HbA1c decrease between groups. From August 2020, participants were randomly allocated to a cohort in a 6:1 DT-to-SC ratio using block randomization with random sequences. This ratio was changed from December 2020 onward to 2:1 after Institutional Review Board approval for a total of 250 DT and 86 SC participants. Seventeen DT and no SC participants discontinued the program without receiving the allocated treatment, leaving 233 DT and 86 SC participants in the intention-to-treat population. Confidentiality of the allocation sequence was maintained until assignments were finalized. Principal investigators enrolled participants.

### Interventions

#### DT Intervention

**Figure 1:**
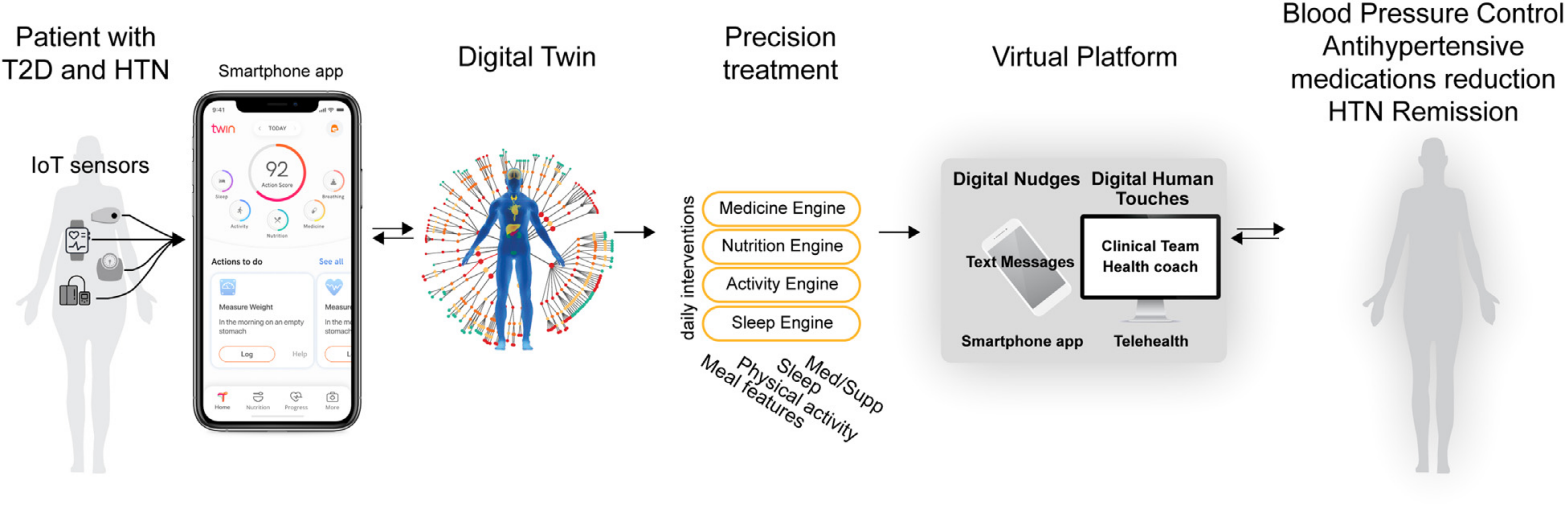
**Personalized Care With Hybridized Nudge-Touch IoT/AI-Driven Digital Twin∗** ∗The Digital Twin platform uses Internet of Things sensors to connect and exchange data with other devices and systems to monitor health status. Once data are analyzed by artificial intelligence, each participant is provided with a series of precision interventions. These recommendations are reviewed by the clinical team and health coaches and then delivered by the App as technological nudges and virtually by health coaches as digital human touches. Additional on-demand counseling and support are provided by health coaches through the App, virtual meetings, or by phone. DT = Digital Twin; HTN = hypertension; IoT = Internet of Things; T2D = type 2 diabetes.

DT intervention is multifaceted, blending advanced technological solutions, such as the DT model and AI-driven analytics, with various patient-centric activities facilitated by coach and medical supervision by physicians (Figure 1). It is important to clarify that the primary driving force of our intervention is the sophisticated technology, particularly the personalized treatment strategies and precise predictive insights generated by the AI. The human element of our intervention, while more minimal in scope, plays a crucial facilitative role. It primarily serves to implement the AI’s recommendations effectively and to add a necessary human touch to the technology-driven process. In the DT group, participants were provided with a continuous glucose monitor (Abbott FreeStyle Libre Pro), sensor watch (Fitbit Charge 2), and smart scale (Powermax BCA-130 Bluetooth Smart Scale) throughout the study period. They were also given a BP meter (TAIDOC TD-3140) and instructions on how to check BP at home. Systemic HTN (systolic blood pressure [SBP] and diastolic blood pressure [DBP]), as measured by a Home Blood Pressure Monitoring system, was evaluated longitudinally for 1 year. Participants were instructed to check their BP twice daily and received daily reminders through the Twin smartphone App (Whole Body Digital Twin), with measurements transmitted to the software securely through a cellular network. Clinical history and laboratory profile were obtained at enrollment. Dietary and other data were gathered using the DT sensors and smartphone App, with all information securely transmitted through a cellular network. At 1-day postenrollment and ensuing 3-month intervals, fasting phlebotomy was performed for biochemical markers. Every quarter, clinic BP measurements were taken by a trained nurse using a BP meter, ensuring the patient had been seated for a minimum of 5 minutes prior to the procedure. The cuff was placed on the subject’s arm so that it was approximately 2 to 3 cm above the elbow joint of the inner arm, with the air tube lying over the brachial artery. The participant’s arm was placed on the table or supported with the palm facing upward, so that the tab of the cuff was placed at the same level of the heart. Triplicate measurements at 1-minute intervals were performed, with the mean of the second and third measurements recorded.

DT platform analyzed dietary intakes from food logs and predicted postprandial glycemic responses (PPGRs) to specific foods based on Internet of Things and AI (machine language algorithms, gradient-boosted decision trees, deep-learning neural networks, and long short-term memory models). Each food was color-coded on the App according to predicted PPGRs for easy recognition: red (avoid), yellow (eat sparingly), or green (eat more). For example, if a subject attempted to log a food with high predicted PPGR, the App would suggest avoidance and provide alternative foods with low predicted PPGRs.

The study had 3 phases: 1) "restricted" phase (90 days) restricting high PPGR foods; 2) “reintroduction” phase (90 days) gradually permitting restricted foods; and 3) “maintenance” phase till the end of the study. Participants ate to satiety. They aimed for 5,000 steps/d initially, then 10,000 steps/d, along with resistance exercises and reduced sedentary time. Sleep duration of >7 to 8 hours and daily deep breathing meditation were encouraged by timely digital nudges. The DT App provided real-time technological nudges, supplemented by remote support from health coaches: 3 times/wk for the first month, then weekly for 2 months, with on-demand assistance thereafter.

Physicians, based on a 7-day average from home BP monitoring, adjusted anti-HTN medication dosages as needed and continued to monitor BP changes. In participants with end organ damage, these adjustments were made with care to maintain beneficial anti-HTN medication while avoiding hypotension.

### Standard of care

In the SC group, enrollment involved collecting clinical histories and laboratory profiles, followed by dietary assessments and consultation insights. Participants adhered to standard T2D and HTN management protocols, integrating lifestyle modifications and pharmacotherapy.^26^ This included nutritional choices such as selecting whole grains, preferring herbs to salt, and choosing items with <400 mg of sodium per serving. The focus was on nonstarchy vegetables, limiting added sugars and processed items, with at least 14 g of fiber per 1,000 kcal, ensuring protein intake of 1 g/kg of body weight, and keeping total fat within 20% to 35% of caloric intake. Guidelines included weight management, moderated alcohol consumption, and smoking cessation recommendations. Dietary practices were complemented by physical activity guidelines: a minimum of 150 minutes of weekly aerobic exercise, resistance training 2 to 3 times weekly on alternating days, and for those over 65, flexibility and balance routines 2 to 3 times per week, aiming to reduce sedentary time for overall health benefits. Biochemical markers were measured via fasting phlebotomy 1-day postenrollment and every 3 months thereafter. Home BP was monitored weekly, coupled with trimonthly physician visits for clinic BP checks, medication adjustments, and consultations for nutritional and exercise guidance based on quarterly biochemical feedback.

### Outcome Measures

Primary outcomes in the clinical trial were HbA1c change and reduction in T2D medications. Secondary outcomes for this analysis included BP change, reduction in anti-HTN medications, HTN remission rates, and microalbuminuria changes. The presence of HTN was determined by an elevated BP (SBP ≥140 mm Hg or DBP ≥90 mm Hg) or use of BP-lowering drugs. Other participants were deemed normotensive. The BP categories were: normal (<130/85 mm Hg); high normal (130-139/85-89 mm Hg); stage 1 (140-159/90-99 mm Hg); and stage 2 (≥160/≥100 mm Hg).^27^ HTN remission was defined as SBP <140 and DBP <90 with no anti-HTN medication.^28^ Normoalbuminuria was defined according to current guidelines by a random urine albumin-creatinine ratio <30 mg/g, microalbuminuria 30 to 300 mg/g, and macroalbuminuria >300 mg/g.^29^

### Statistical Analysis

Continuous or quantitative variables were presented as mean ± SD. Categorical or nominal data measurements were presented in count (%). The Shapiro-Wilk test was used to detect the normality of data. The paired *t*-test was used to detect significance between study parameters in the cohorts measured on 2 occasions. Independent sample *t*-test was used to compare the continuous or quantitative variables between the groups. A 2-sided *P* < 0.05 was considered statistically significant. A linear mixed-effects model analyzed BP changes across trial phases, accounting for baseline covariates and interindividual variability (Supplemental Appendix).

## Results

### Participants

**Figure 2:**
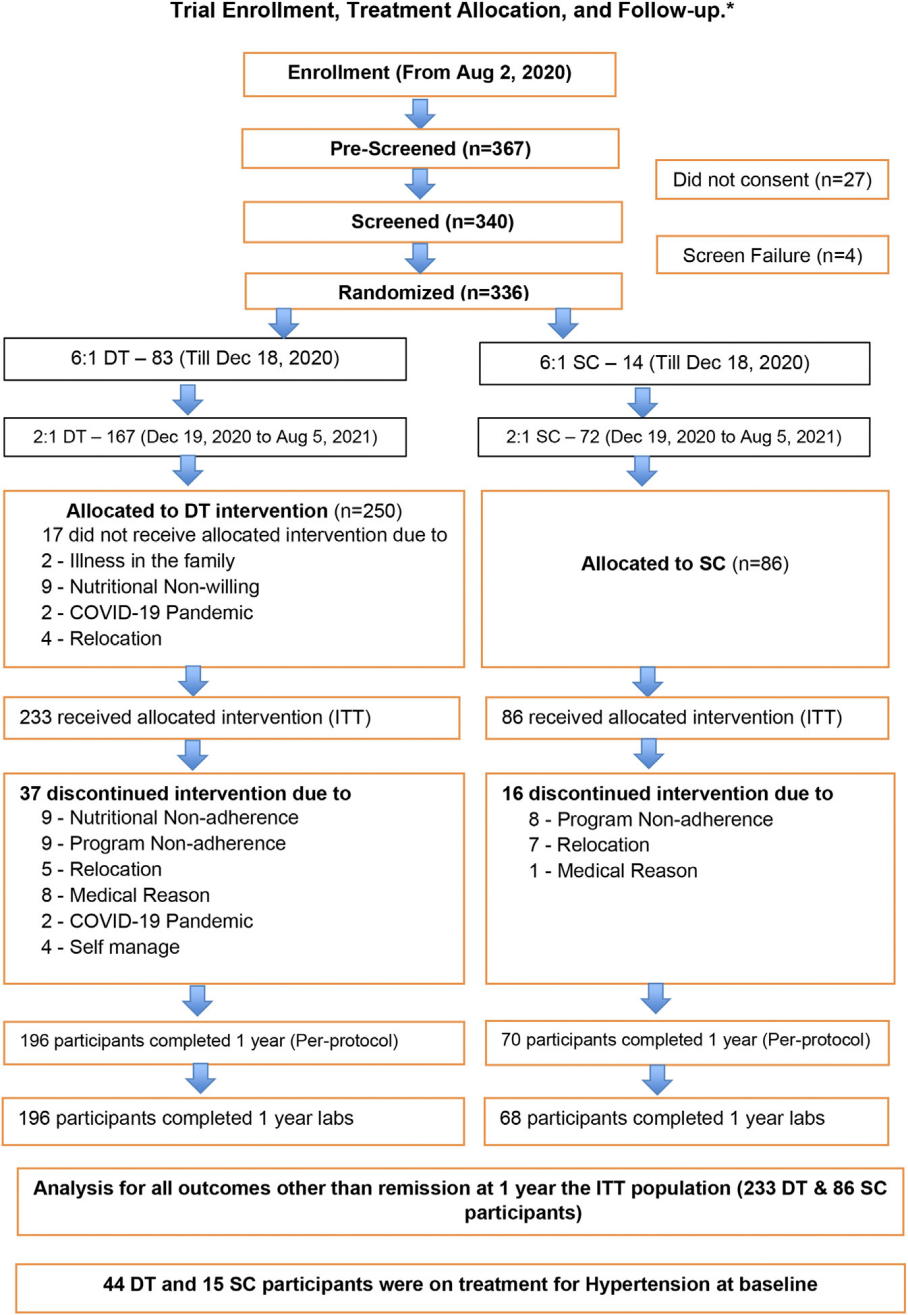
**Participant Flowchart** ∗37 Digital Twin participants and 16 standard of care participants were lost to follow-up by 1 year. We conducted the analysis using the “last observation carried forward” to handle missing observations for these participants. DT = Digital Twin; ITT = intention-to-treat; SC = standard of care.

The study sample consisted of 319 enrolled participants randomized to the DT (N = 233) or SC (N = 86) groups (Figure 2). At baseline, participants had a mean age of 43.6 ± 8.6 years and duration of diabetes of 3.8 ± 2.6 years; 44 in the DT and 15 in the SC group were on treatment for HTN at baseline.

Baseline characteristics for both DT and SC groups are shown in Table 1.

**Table 1:** Baseline Characteristics of Study Participants

	DT (n = 233)	SC (n = 86)
Age, y	43.6 ± 8.6	51.8 ± 10.5
Sex		
Male	195 (83.7)	40 (46.5)
Female	38 (16.3)	46 (53.5)
Weight, kg	78.4 ± 14.5	71.7 ± 13.1
BMI, kg/m^2^	27.2 ± 4.4	28.1 ± 4.4
Waist circumference, cm	97.6 ± 11.2	95.0 ± 10.3
HbA1c, %	9 ± 1.9	8.4 ± 1.9
Mean number of T2D medications	1.8 ± 1.1	1.8 ± 1.1
Fasting insulin, mean (SD), μU/mL	13.5 ± 9.5	14 ± 9.6
HOMA2-IR	1.9 ± 0.9	1.9 ± 0.9
Number of participants on antihypertensive medications (%)	44 (18.9%)	15 (17.4%)
Systolic blood pressure, mm Hg	127.5 ± 11.3	133.7 ± 17.2
Diastolic blood pressure, mm Hg	84.8 ± 7.3	87.0 ± 10.4
Number. of antihypertensive medications	0.2 ± 0.5	0.2 ± 0.4
MCIR, mg/g	36.6 ± 97.2	29.5 ± 58.6

Values are mean ± SD or n (%).

BMI = body mass index; DT = digital twin; MICR = microalbumin-to-creatinine ratio; HOMA2-IR = homeostatic model assessment-2 for insulin-resistance; SC = standard of care; T2D = type 2 diabetes.

### Systolic and Diastolic Blood Pressure

**Figure 3:**
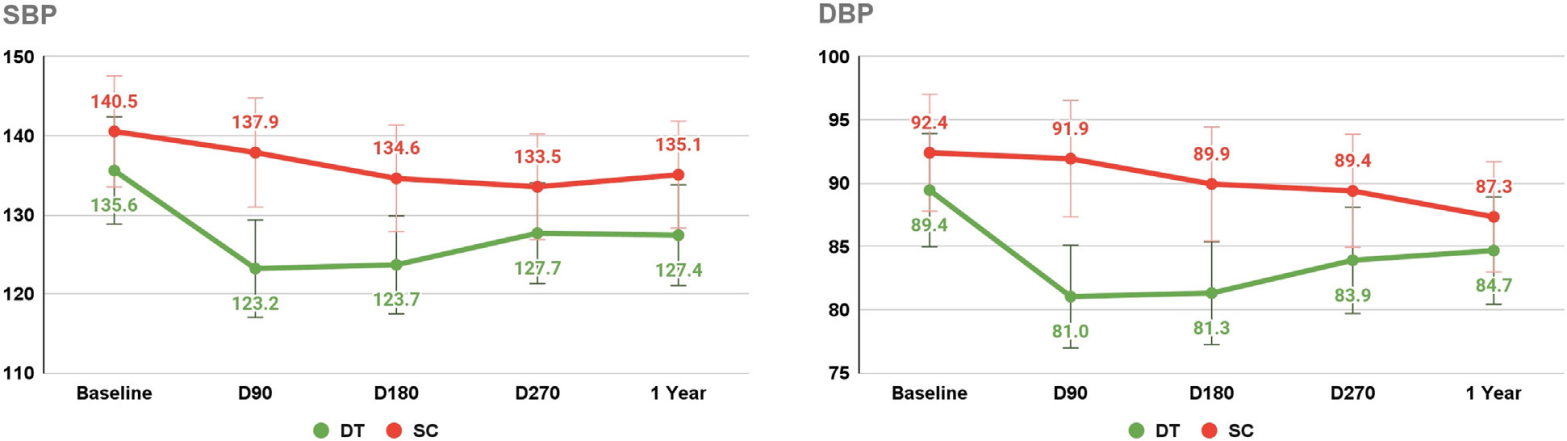
**Serial Changes in Systolic and Diastolic Blood Pressure After 1 Year Among the Hypertension Subgroup in the DIGITAL Twin Intervention and the Standard of Care Control Groups** DBP = diastolic blood pressure; DT = digital twin; SBP = systolic blood pressure; SC = standard of care.

Table 2 details the baseline to 1-year SBP and DBP changes in both DT and SC groups, revealing significant differences. The DT group showed a greater SBP reduction at 1 year compared to SC (−7.6 [12.2] mm Hg vs −3.2 [15.2] mm Hg; *P* = 0.007), alongside greater DBP reduction (−4.3 [8.4] mm Hg vs −2.2 [9.1] mm Hg; *P* = 0.046). In participants with baseline HTN, the DT subgroup achieved greater SBP reduction at 1 year than the SC subgroup (−9.9 [13.1] mm Hg vs −5.5 [18.3] mm Hg; *P* = 0.316). DBP reduction was also greater in the DT subgroup compared to SC at 1 year (−6.3 [8.6] mm Hg vs −5.1 [12.3] mm Hg; *P* = 0.679) (Figure 3).

**Table 2:** Changes in Hypertension Outcome Measures After 1 Year Among T2D Participants in the DT Intervention and the SC Control Groups^a^

Outcome Measures	Treatment Group	N				*P* Value		
			Baseline	1 Year	Change	Within Group^a^	Change^b^	Between Groups at 1 Year^b^
Systolic blood pressure, mm Hg	DT	233	127.4 ± 11.3	119.8 ± 11.8	7.6 ± 12.1	**<0.001**	**0.008**	**<0.001**
	SC	86	134.1 ± 17.2	130.9 ± 13.6	3.2 ± 15.2	0.06		
Diastolic blood pressure, mm Hg	DT	233	84.9 ± 7.2	80.5 ± 8.7	4.3 ± 8.4	**<0.001**	**0.047**	**<0.001**
	SC	86	87.3 ± 10.4	85.1 ± 7.1	2.2 ± 9.1	**0.032**		
Number of participants on antihypertensive medications	DT	233	44 (18.9%)	14 (6.0%)	30 (12.8%)	-	-	**<0.001**
	SC	86	15 (17.4%)	15 (17.4%)	0 (0%)			
Number of antihypertensive medications	DT	233	0.2 ± 0.5	0.1 ± 0.4	0.1 ± 0.5	**0.018**	0.102	0.076
	SC	86	0.2 ± 0.4	0.2 ± 0.4	−0.0 ± 0.1	0.32		

Values are N, mean ± SD, or n (%). Values in **bold** are significant with *P* < 0.05.

DT = digital twin; SC = standard of care; T2D = type 2 diabetes.

a Paired *t*-test,

b Independent sample *t*-test.

Using mixed-effect model (Table 3), DT group saw decrease in SBP by 10.14 and DBP by 6.69 during restrictive phase. Over 1 year, DT group's adjusted changes were 7.51 for SBP and 4.49 for DBP. Site effect is not significant (Supplemental Appendix).

**Table 3:** Impact of Different Phases on Blood Pressure Measurements in DT and SC Groups Obtained by Mixed-Effects Model: Changes in Systolic and Diastolic Blood Pressure Before and After Adjustment for Covariates^a^

Outcome Measures	Treatment Group	Mean at Baseline (mm Hg)	Model Change From Baseline to 1 Year (mm Hg)	Adjusted Change (mm Hg)	Adjusted Change in Restrictive Phase (mm Hg)	Adjusted Change in Reintroduction Phase (mm Hg)	Adjusted Change in Maintenance Phase (mm Hg)	Site Effect (*P* Value)
Systolic blood pressure (mm Hg)	DT	127.4	7.82	7.51	−10.14	0.43	2.2	0.923
	SC	134.1	2.09	2.1	NA	NA	NA	0.255
Diastolic blood pressure (mm Hg)	DT	84.9	4.66	4.49	−6.69	0.46	1.74	0.355
	SC	87.3	1.95	1.97	NA	NA	NA	0.491

DT = digital twin; NA = not applicable as there is no phase differences in the SC group; SC = standard of care.

a Data derived from mixed-effects modeling of longitudinal values of systolic and diastolic blood pressure.

### HTN Categories

Changes in HTN categories were assessed in the DT and SC groups (Central Illustration).

**Central Illustration:**
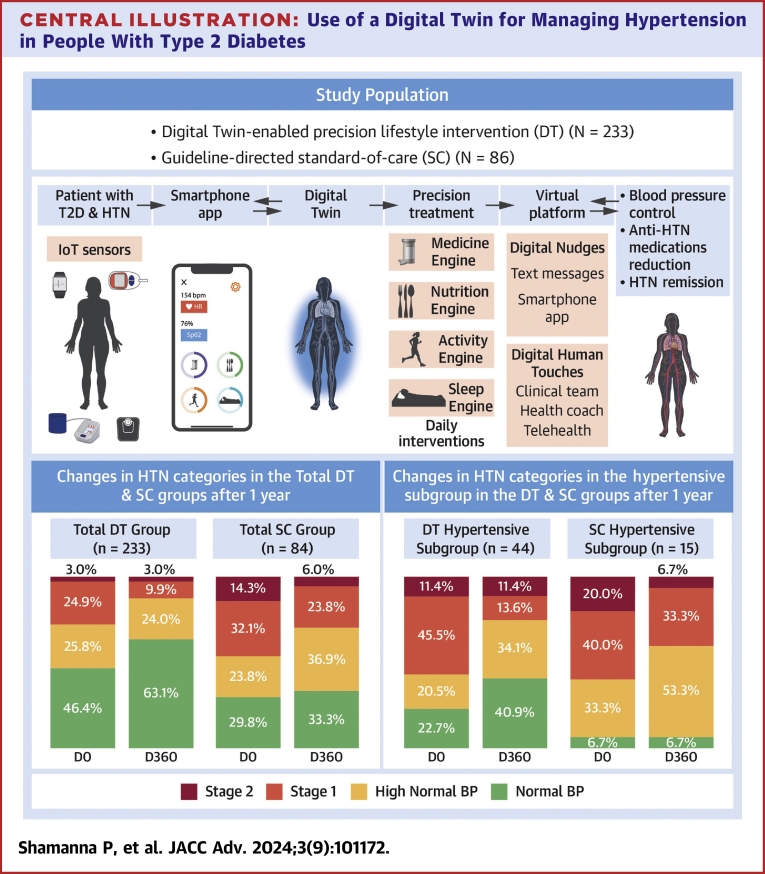
**Use of a Digital Twin for Managing Hypertension in People With Type 2 Diabetes** The blood pressure categories were: normal (<130/85 mm Hg); high normal (130-139/85-89 mm Hg); stage 1 (140-159/90-99 mm Hg); and stage 2 (≥160/≥100 mm Hg). BP = blood pressure; DT = digital twin; HTN = hypertension; T2D = type 2 diabetes.

#### DT group

In the DT group (n = 233), there was a significant improvement in BP management from baseline to 1 year. The number of participants with normal BP increased from 108 (46.4%) to 147 (63.1%). Those in the high normal BP category decreased from 60 (25.8%) to 56 (24.0%). A marked reduction was seen in stage 1 HTN, dropping from 58 (24.9%) to 23 (9.9%). Stage 2 HTN cases remained constant at 7 (3.0%).

#### SC group

For the SC group (n = 84), changes in BP levels from baseline to 1 year were less dramatic. The number of participants with normal BP slightly increased from 25 (30%) to 28 (33%). High normal BP cases rose from 20 (24%) to 31 (37%). Stage 1 HTN decreased from 27 (32%) to 20 (24%). Stage 2 HTN cases saw a reduction from 12 (14%) to 5 (6%).

In the DT group, more participants achieved normotension than in the SC group (147 [63.1%] vs 28 [33.3%]), with a relative risk (RR) of 0.6 (95% CI: 0.4-0.7; *P* < 0.00001). Among high normal BP category, fewer participants in the DT group were affected compared to the SC group (56 [24.0%] vs 31 [37%]), RR: 0.6 (95% CI: 0.4-0.9; *P* = 0.03). For stage 1 HTN, fewer participants in the DT group were affected compared to the SC group (23 [9.9%] vs 20 [24%]), with an RR of 0.4 (95% CI: 0.2-0.7; *P* = 0.002). For stage 2 HTN, fewer participants in the DT group were affected compared to the SC group (7 [3.0%] vs 5 [6.0%]), RR: 0.5 (95% CI: 0.2-1.5; *P* = 0.313).

#### DT hypertensive subgroup

In the DT hypertensive subgroup (n = 44), there were notable improvements in BP categories from baseline to 1 year. The proportion of participants with normal BP increased from 10 (22.7%) to 18 (40.9%). Those classified as high normal BP rose from 9 (20.5%) to 15 (34.1%). A significant decrease was observed in stage 1 HTN, falling from 20 (45.5%) to 6 (13.6%). Stage 2 HTN remained stable at 5 (11.4%).

#### SC hypertensive subgroup

In the SC hypertensive subgroup (n = 15), changes in BP categories from baseline to 1 year were more moderate. The number of participants with normal BP remained constant at 1 (7%). High normal BP cases increased from 5 (33%) to 8 (53%). Stage 1 HTN decreased from 6 (40%) to 5 (33%). Stage 2 HTN cases reduced from 3 (20%) to 1 (7%).

In hypertensive subgroups, more DT participants achieved normotension than SC (18 [40.9%] vs 1 [6.7%]), RR: 0.6 (95% CI: 0.4-0.8; *P* = 0.0009). Among high normal BP, fewer DT participants were affected compared to SC (18 [40.9%] vs 8 [53%]), RR: 0.6 (95% CI: 0.2-1.2; *P* = 0.310). For stage 1 HTN, fewer DT participants were affected compared to SC (6 [13.6%] vs 5 [33%]), RR: 0.4 (95% CI: 0.1-1.1; *P* = 0.112). For stage 2 HTN, fewer DT participants were affected compared to SC (5 [11.4%] vs 1 [7.0%]), RR: 0.5 (95% CI: 0.2-1.5; *P* = 1.000) (Central Illustration).

### Antihypertensive Medications

**Figure 4:**
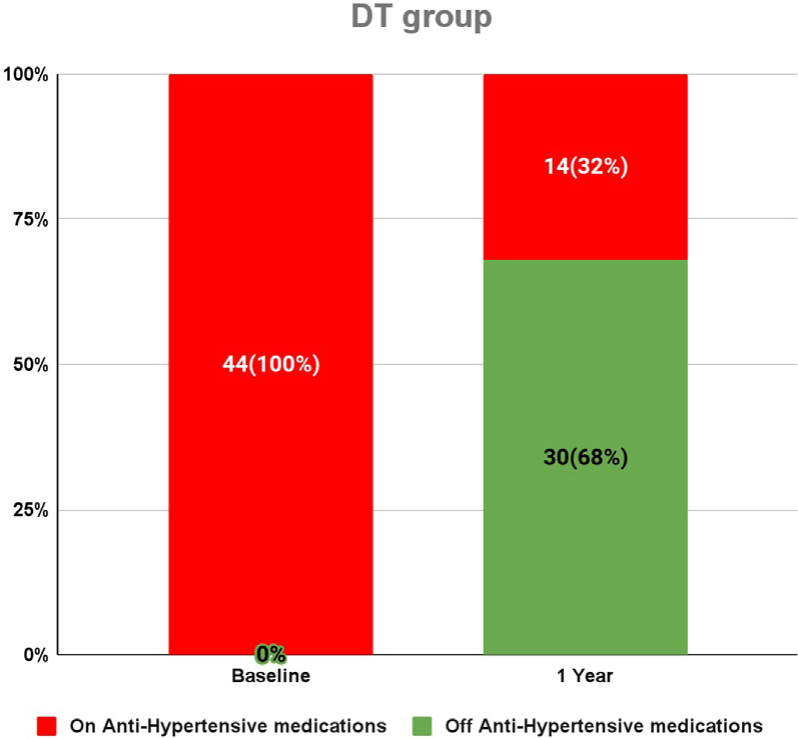
**Number of Participants on Antihypertensive Use at Baseline and 1 Year** Hypertension remission was defined as systolic blood pressure <140 and diastolic blood pressure <90 and no antihypertensive medication. Out of 44 digital twin participants, 30 (68.2%) digital twin participants did not need antihypertensive medication usage at D360. Of these 22 (50%) achieved remission by the above definition. In the standard of care group, 15 (17.4%) participants used antihypertensives at baseline (14 on 1 medication/d; 1 on 2 or more medications/d). All 15 standard of care participants continued antihypertensive use. DT = digital twin.

After 1 year, the DT intervention decreased anti-HTN medication usage among the HTN subgroup. Initially, 44 out of 233 participants (18.9%) in the DT group were on anti-HTN medications, with 32 taking 1 daily and 12 on 2 or more daily. At the 1-year mark, only 14 of these 44 participants (31.8%) continued anti-HTN medications (8 on 1 daily; 6 on 2 or more daily), while 30 participants (68.2%) discontinued their medication. At baseline in the SC group, 15 out of 86 participants (17.4%) were on anti-HTN medications, with 14 taking 1 daily and 1 on 2 or more daily. After 1 year, all 15 participants maintained their anti-HTN medication usage at the same levels as at baseline. A higher proportion of the DT hypertensive subgroup discontinued anti-HTN medications compared to the SC hypertensive subgroup (RR: 0.3 for medication reduction; 95% CI: 0.2-0.5; *P* < 0.0001) (Figure 4).

### Hypertension

At 1 year, DT intervention achieved remission in participants with HTN (Figure 4). In the DT group, 44 (18.9%) participants had HTN at baseline. At 1 year, 22 (50%) participants achieved remission; 8 (18.2%) still had high normal BP without anti-HTN medication; and 14 (31.8%) remained on antiHTN medication). In the SC group, 15 (17.4%) participants had HTN at baseline, with none having HTN remission at 1 year. More participants in the DT hypertensive subgroup achieved HTN remission compared to the SC group (22 [50%] vs 0 [0%], respectively; RR: 0.5; 95% CI: 0.3-0.7; *P* < 0.0001) (Figure 4).

The status of microalbuminuria was evaluated for the entire cohort and specifically within the hypertensive subgroups, based on assessments conducted at baseline and after 1 year for participants in the DT and SC groups. After 1 year in the DT group, the incidence of microalbuminuria reduced from 47 (21.1%) to 17 (7.6%) overall, and specifically within the hypertensive subgroup, it decreased from 16 (38.1%) to 4 (9.5%). At 1 year in the DT group, the incidence of normoalbuminuria increased from 176 (78.9%) to 206 (92.4%) overall, and specifically within the hypertensive subgroup, it increased from 26 (61.9%) to 38 (90.5%). After 1 year in the SC group, the incidence of microalbuminuria remained stable at 14 (16.9%) overall, while in the hypertensive subgroup, it increased from 0 (0%) to 2 (13.3%). At 1 year in the SC group, the incidence of normoalbuminuria remained the same at 69 (83.1%) overall, while in the hypertensive subgroup, it decreased from 15 (100%) to 13 (86.7%). More participants in the DT group achieved normoalbuminuria compared to the SC group (206 [92.4%] vs 69 [83.1%], respectively; *P* = 0.02).

## Discussion

In this study, participants with T2D and HTN showed significant improvements in SBP and DBP, reduction of anti-HTN medications, significant HTN remission rates, and amelioration of microalbuminuria with DT intervention compared to SC. The decrease in anti-HTN pill burden improves patient adherence and can be guided by supervised deprescribing strategies.^24^^,^^30^^,^^31^ In parallel with these benefits, we found significant improvements in HbA1c, weight, body mass index, and waist circumference in the DT group compared to the SC group, as reported elsewhere.^19^ Albuminuria is a known predictor of all-cause and cardiovascular mortality,^32^^,^^33^ and in patients with T2D, it is a characteristic finding of renal disease.^34^^,^^35^ Our results indicate significant improvements in microalbumin creatinine ratio in the DT group compared to the SC group. The number of participants with normoalbuminuria increased among the total DT and the DT hypertensive subgroup.

The SC hypertensive subgroup maintained their anti-HTN medication usage at the same levels as at baseline. In contrast, hypertensive participants in the DT group with improvement in the metabolic and adiposity parameters had a decrease in the need for anti-HTN medications, and 50% of participants were able to maintain normal BP without anti-HTN medications and achieved remission of HTN.

The use of DT technology to create personalized nutrition, activity, and sleep for participants with chronic diseases such as T2D and HTN represents a significant optimization step. This technology can accurately predict PPGRs by employing continuous glucose monitor sensors, additional sensor information (activity, heart rate, and weight) via the Internet of Things, and AI (eg, machine learning algorithms. As a result, nutrition, activity, and sleep behavior are optimized to achieve superior glycemic control and consequently ameliorate hyperinsulinemia. The reduction of hyperinsulinemia leads to the mobilization and oxidation of stored fat, leading to weight loss and insulin resistance reduction, as seen by the reduction of HOMA2-IR (homeostatic model assessment-2 for insulin-resistance).^19^^,^^36^ This conglomeration of changes in weight, adiposity, and insulin resistance contributes to BP reduction.

Indicators of inflammation such as HOMA2IR (homeostatic model assessment-2 for insulin-resistance), Ferritin, and white blood cell count show significant improvement in the DT group and could have a contributory role in the reduction of BP.^19^^,^^37^

The effectiveness of the DT intervention in our study can be attributed to its ability to mitigate glucotoxicity, inflammation, insulin resistance, and obesity, which are key factors in managing diabetes and HTN.^19^ The DT approach uniquely fosters comprehensive behavioral changes through digital nudges, promoting adherence to AI-driven, personalized recommendations for diet, sleep, exercise, and reduced sedentary behavior. Unlike programs that focus on drastic caloric or carbohydrate restrictions, the DT model emphasizes personalized food choices based on the lowest predicted PPGR, helping to avoid decreased basal metabolic rates and the subsequent rebound weight gain often seen with other interventions.^38^

The strengths of this study are related to the novel AI-enabled DT intervention and prior demonstration of significant T2D remission in the cohort. The limitations of this study are related to problems of generalizability due to ethnocultural factors and the relatively small HTN subgroup. Longer follow-up durations and larger sample sizes in future studies can generate more robust evidence to improve BP in patients with T2D. Our trial's randomization aimed to minimize selection and performance biases by distributing technology predispositions across DT and SC arms, enabling rigorous outcome comparisons, though not fully eliminating all biases. In the DT group, participants measured their BP twice daily, enhancing management and awareness. Conversely, the SC group measured BP at home twice per week, adhering to guidelines and maintaining the study's control structure, while preserving the trial's integrity.

## Conclusions

Various lifestyle interventions based on diet, physical activity, and sleep modifications can promote HTN remission. The AI-enabled DT technology is a lifestyle medicine intervention that results in significant improvements in cardiometabolic risk factors, including HTN. Future long-term research on personalized nutritional management in T2D can help generate large-scale generalizable data, benefitting patient interests at large.

PERSPECTIVES **COMPETENCY IN MEDICAL KNOWLEDGE:** The study reveals Digital Twin technology's superior efficacy in managing HTN among people with type 2 diabetes, showing significant blood pressure reductions, less dependence on anti-HTN medication, and more HTN remission.

**TRANSLATIONAL OUTLOOK 1:** Findings advocate a new approach in HTN care for type 2 diabetes. Future studies should assess this technology's effectiveness across various populations.

**TRANSLATIONAL OUTLOOK 2:** Further large-scale long-term trials are needed to validate Digital Twin technology in hypertension management, with a focus on understanding underlying mechanisms for personalized therapies.

## Funding Support and Author Disclosures

Dr Joshi is a scientist at Twin Health Inc; has received consulting fees from Franco Indian, Biocon, Zydus Cadila, Glenmark, Torrent, and Marico; and has received speaker honoraria or has served on the advisory board of MSD, Novo Nordisk, Sanofi, Boehringer Ingelheim, Abbott, AstraZeneca, Serdia, Alkem, Lupin, Bayer Zydus, and USV. Drs Shamanna, Keshavamurthy, and Shah are employees of Twin Health Inc. Drs Mechanick, Dharmalingam, Vadavi, and Samajdar are members of the Advisory Board of Twin Health Inc. Dr Mechanick received speaker honoraria and is on advisory board for Abbott Nutrition.
