# Supplementary material for: Digital Twin in Managing Hypertension Among People With Type 2 Diabetes: 1-Year Randomized Controlled Trial
Source: JACC Adv. 2024 Aug 14;3(9):101172. doi: 10.1016/j.jacadv.2024.101172 (PMC11450914; doi:10.1016/j.jacadv.2024.101172)
Supplement: Supplementary Appendix [file mmc1.docx]

**SUPPLEMENTAL APPENDIX**

**Methods**

**Mixed-effects model**

A linear mixed-effects model analyzed BP changes across trial phases, accounting for baseline covariates and inter-individual variability

**Model Specification:**

1. Type of Model: Linear Mixed-Effects Model
2. Dependent Variable: Blood pressure outcome (continuous variable, e.g., systolic blood pressure)
3. Fixed Effects:

- Baseline clinical parameters (e.g., age, weight, BMI)
- Treatment factors (e.g., medication use, lifestyle interventions)
- Demographic variables (e.g., gender, ethnicity)

1. Random Effects:

- Subject-specific intercepts: To account for the variability in blood pressure outcomes between individuals that is not explained by the fixed effects.
- Time-specific slopes: To capture individual differences in the rate of change of blood pressure over time.

1. Covariance Structure:

- A random intercept and slope model with an unstructured covariance matrix were assumed to allow for flexibility in estimating the relationship between time and blood pressure outcomes without imposing constraints on the covariance among the random effects.

**Verification of Modeling Assumptions:**

1. **Linearity**:
   - The relationship between predictors and blood pressure was assessed using scatter plots and residuals to ensure a linear relationship.
2. **Normality of Residuals**:
   - Residuals were examined using Q-Q plots and histograms to check for normal distribution.
3. **Independence of Errors**:
   - Assumed independence of residuals for different subjects. This was checked by examining residuals across different time points and subjects to ensure no autocorrelation.
4. **Homoscedasticity**:
   - Plots of residuals versus fitted values were used to check for constant variance across different levels of the predictors.
5. **Multicollinearity**:
   - Variance Inflation Factors (VIF) were calculated to ensure there was no problematic multicollinearity among the fixed effects.
6. **Random Effects Distribution**:
   - Random effects were assumed to be normally distributed. This was checked using Q-Q plots of the random effect estimates.
7. **Model Fit**:
   - The model fit was assessed using the Akaike Information Criterion (AIC) and Bayesian Information Criterion (BIC). Lower values indicated a better fit.
   - Conditional and marginal R-squared values were computed to evaluate the proportion of variance explained by the fixed and random effects.
8. **Goodness of Fit**:
   - Residual plots were used to visually assess model fit. Deviations from the assumptions were checked and model adjustments were made if necessary.

These steps ensured that the linear mixed-effects model provided a robust framework for understanding how baseline parameters influenced blood pressure outcomes over time while accounting for individual variability and time-related changes.

**Results**

**Analysis of Systolic Blood Pressure Changes Across Trial Phases:**

**Fixed Effects:**

1. **Trial Phases**:
   - **Restrictive Phase**:
     - There was a significant decrease in systolic blood pressure during the restrictive phase compared to baseline. The average reduction was approximately 10 mmHg (p < 0.01).
   - **Reintroduction Phase**:
     - During the reintroduction phase, systolic blood pressure slightly increased compared to the restrictive phase, but the increase was not statistically significant (p > 0.05). The change suggested stabilization rather than regression.
   - **Maintenance Phase**:
     - In the maintenance phase, systolic blood pressure showed a slight increase compared to the reintroduction phase but remained significantly lower than the baseline. The average reduction from baseline was about 7 mmHg (p < 0.05).
2. **Baseline Covariates**:
   - **Age**: Older participants tended to have smaller reductions in systolic blood pressure across all phases (p < 0.05).
   - **Baseline Systolic Blood Pressure**: Higher initial systolic blood pressure was associated with greater reductions (p < 0.01), suggesting that those with more elevated baseline values experienced more significant improvements.
   - **Gender**: Males had a slightly greater reduction in systolic blood pressure compared to females, though the difference was not statistically significant (p > 0.05).
3. **Interaction Terms**:
   - The interaction between trial phases and baseline systolic blood pressure was significant (p < 0.01), indicating that the effect of the trial phase on systolic blood pressure varied depending on initial blood pressure levels.

**Random Effects:**

- **Subject-Specific Intercepts**:
  - There was considerable variability in baseline systolic blood pressure levels among individuals, with standard deviations reflecting substantial inter-individual differences.
- **Subject-Specific Slopes**:
  - Variability in the change in systolic blood pressure across the different trial phases was observed, indicating that individuals responded differently to each phase of the trial.

**Covariance Structure:**

- The unstructured covariance matrix indicated significant correlations between the random intercepts and slopes, suggesting that the initial systolic blood pressure level influenced the magnitude of change across the phases.

**Model Fit:**

- The model fit well, with the Akaike Information Criterion (AIC) and Bayesian Information Criterion (BIC) indicating better performance compared to models without random effects.
- Conditional R-squared values suggested that about 70% of the variance in systolic blood pressure changes was explained by the model's fixed and random effects.

**Interpretation:**

The results indicated that the restrictive phase of the trial was most effective in reducing systolic blood pressure, with sustained effects observed during the reintroduction and maintenance phases. Individual variability played a significant role, highlighting the importance of personalized approaches in managing blood pressure.

These findings underscore the effectiveness of a phased approach in controlling systolic blood pressure and emphasize the need for ongoing management to maintain the benefits achieved during the initial restrictive phase.

**Supplemental Table 1. Blood Pressure Changes Over One Year Across Different Sites in the DT group**

| **Site #** | **Baseline SBP**  **(mmHg)** | **1 year SBP**  **(mmHg)** | **Change in SBP between baseline and 1 year**  **(mmHg)** | **Change in SBP  p-value^$^** | **Change in SBP between sites**  **p-value^∍^** | **Baseline DBP**  **(mmHg)** | **1 year DBP**  **(mmHg)** | **Change in DBP between baseline**  **and 1 year**  **(mmHg)** | **Change in DBP  p-value^$^** | **Change in DBP between sites p-value^∍^** |
| --- | --- | --- | --- | --- | --- | --- | --- | --- | --- | --- |
| 1 | 127.68 (11.63) | 120.33 (12.15) | -7.34 (12.22) | **<0.001** | 0.213 | 85.12 (7.50) | 80.77 (8.78) | -4.35 (8.74) | **<0.001** | 0.698 |
| 2 | 123.40 (10.63) | 117.03 (9.00) | -6.37 (12.75) | 0.097 |  | 82.38 (7.58) | 79.08 (8.22) | -3.31 (7.81) | 0.153 |  |
| 3 | 130.53 (11.56) | 118.27 (9.51) | -12.25 (12.42) | **<0.001** |  | 85.96 (5.58) | 80.03 (7.83) | -5.92 (7.18) | **<0.001** |  |
| 4 | 124.87 (8.51) | 119.45 (12.61) | -5.42 (10.64) | **0.020** |  | 83.28 (6.33) | 80.00 (9.35) | -3.28 (7.30) | **0.038** |  |

* Data are mean (SD), ^$^ paired t-test, ^∍^ ANOVA. † Figures in **bold** are significant with p-value <0.05. Abbreviations: DT – digital twin; DBP – diastolic blood pressure; SBP – systolic blood pressure; SC – standard of care.
